# Rodent hole detection in a typical steppe ecosystem using UAS and deep learning

**DOI:** 10.3389/fpls.2022.992789

**Published:** 2022-12-16

**Authors:** Mingzhu Du, Dawei Wang, Shengping Liu, Chunyang Lv, Yeping Zhu

**Affiliations:** ^1^ Agricultural Information Institute, Chinese Academy of Agricultural Sciences, Beijing, China; ^2^ Key Laboratory of Agricultural Blockchain Application, Ministry of Agriculture and Rural Affairs, Beijing, China; ^3^ State Key Laboratory for Biology of Plant Diseases and Insect Pests, Institute of Plant Protection, Chinese Academy of Agricultural Sciences, Beijing, China; ^4^ Institute of Grassland Research, Chinese Academy of Agricultural Sciences, Key Laboratory of Biohazard Monitoring and Green Prevention and Control in Artificial Grassland, Ministry of Agriculture and Rural Affairs, Hohhot, China

**Keywords:** rodent monitoring, mouse hole detection, grassland protection, unmanned aircraft vehicle (UAV), object detection

## Abstract

**Introduction:**

Rodent outbreak is the main biological disaster in grassland ecosystems. Traditional rodent damage monitoring approaches mainly depend on costly field surveys, e.g., rodent trapping or hole counting. Integrating an unmanned aircraft system (UAS) image acquisition platform and deep learning (DL) provides a great opportunity to realize efficient large-scale rodent damage monitoring and early-stage diagnosis. As the major rodent species in Inner Mongolia, Brandt’s voles (BV) (*Lasiopodomys brandtii*) have markedly small holes, which are difficult to identify regarding various seasonal noises in this typical steppe ecosystem.

**Methods:**

In this study, we proposed a novel UAS-DL-based framework for BV hole detection in two representative seasons. We also established the first bi-seasonal UAS image datasets for rodent hole detection. Three two-stage (Faster R-CNN, R-FCN, and Cascade R-CNN) and three one-stage (SSD, RetinaNet, and YOLOv4) object detection DL models were investigated from three perspectives: accuracy, running speed, and generalizability.

**Results:**

Experimental results revealed that: 1) Faster R-CNN and YOLOv4 are the most accurate models; 2) SSD and YOLOv4 are the fastest; 3) Faster R-CNN and YOLOv4 have the most consistent performance across two different seasons.

**Discussion:**

The integration of UAS and DL techniques was demonstrated to utilize automatic, accurate, and efficient BV hole detection in a typical steppe ecosystem. The proposed method has a great potential for large-scale multi-seasonal rodent damage monitoring.

## 1 Introduction

Rodent infestation is one of the main biological hazards that seriously affect the health of grassland ecosystems (Liu, 2022). In grassland ecosystems in Mongolian Plateau, Brandt’s vole (BV, *Lasiopodomys brandtii*) is the major pest, which is a small, seasonal breeding rodent species living in social groups and digging complex burrow systems with up to approximately 5,616 holes/ha in high-density areas (Zhong et al., 1999). Dense BV holes accelerated erosion and desertification in grasslands, resulting in mass herbage and forage loss in Inner Mongolia (Zhang and Wang, 1998). In addition, BV is also the intermediate host for many severe human infectious diseases (Brown and Laco, 2015). Accurate and rapid detection of BV holes is an urgent need to evaluate the rodent population density for better ecosystem and human health protection.

Traditionally, grassland rodent hole detection mainly relied on field surveys. Field surveys can straightforwardly obtain rodent information but are time-consuming and labor-intensive (Li et al., 2016a; Li et al., 2016b; Wang et al., 2019). Recently, UAS, which has a millimeter-level spatial resolution and can quickly collect multi-scale, multi-temporal images in real-time, has emerged as a promising alternative for rodent hole investigation. Nesting traces (burrows, mounds, tunnels, etc.) of many rodent species, i.e., Yellow Stepped Vole (*Eolagurus luteus*) (Xuan et al., 2015), Great Gerbil (*Rhombomys optimus*) (Ma et al., 2018a; Ma et al., 2018b) and Plateau Pika (*Ochotona curzoniae*) (Guo et al., 2017), were able to be identified through manual interpretation. Nevertheless, manual interpretation is still labor-intensive and time-consuming in regard to the large number of images generated by UAS. To solve this problem, scholars have tried to apply various man-machine interaction algorithms to count rodent holes in UAS imagery, such as maximum likelihood classification (Wen et al., 2018), object-oriented classification (Zhou et al., 2018; Sun et al., 2019), support vector machine (SVM) (Heydari et al., 2020), and Sobel filter (Zhao et al., 2016). All these methods have limitations in effectiveness and efficiency when compared with deep convolutional neural networks.

Deep learning (DL) shows great potential in BV hole detection, benefiting from the application of automatic deep convolutional feature extraction (LeCun et al., 2015; Mountrakis et al., 2018; Liu et al., 2020). Recent studies using DL and UAS images have been widely applied to small object detection, such as detecting birds (Hong et al., 2019) and mammals in the wild (Kellenberger et al., 2018; Peng et al., 2020; Jintasuttisak et al., 2022), identifying weeds (Etienne et al., 2021), counting small plants (Oh et al., 2020), and extracting vehicles (Xu et al., 2016, Amato et al., 2019). So far, four previous studies have employed UAS images and DL in rodent hole detection. Cui et al. (2020) identified large gerbil holes (6-12 cm in diameter) in desert forests using You Only Look Once (YOLO)v3 and YOLOv3-tiny. Zhou et al. (2021) detected holes of Plateau Pika (*Ochotona curzoniae*) with a diameter of 8-12 cm, which is a medium-sized rodent, using a Mask Region-based Convolutional Neural Network (R-CNN). Wan et al. (2021) successfully detected grassland rat holes (unspecified species) using R-CNN and improved Single Shot MultiBox Detector (SSD). Ezzy et al. (2021) detected Levant voles burrows (2.5-7.5 cm in diameter) in farmlands and found that YOLOv3 provided relatively accurate and robust results. Previous studies explored various algorithms to detect different rodent holes under various environments. However, no specific one has focused on small-sized rodent holes, e.g., BV holes (4-6 cm in diameter), in a complex typical steppe ecosystem.

The following issues are encountered in BV hole detection in a typical steppe ecosystem. First, the characteristics of BV make hole detection challenging. BV is small-sized, making them much more difficult to be detected from UAS images than other rodent holes. In addition, unlike other rodent species, e.g., Gerbillinae, BV digs holes in a different way that would not result in obvious excavated soil around holes. Visual features of other rodent holes cannot be utilized directly in BV holes. Furthermore, the typical steppe ecosystem, which is the main habitat of BV, has many factors that can impede detection. Animal droppings, hoofprints, and, most importantly, shadows and shades of grass and rocks make BV holes difficult to visually identify from images or even in the field. Different occlusion and illumination conditions at different times and seasons will lead to various spectral and geometric features of BV holes, thus requiring a robust detection method for different seasons, which refers to better generalizability. For example, more lush grass in summer will result in more occlusion in hole observations than in winter, thus making the detection more difficult. To sum up, BV hole detection in a typical steppe ecosystem requires a new dataset and a suitable detection method that can overcome the abovementioned issues in different seasons.

On account of the practical problems, this study aims to develop a specific UAS image dataset and a cost-effective and robust DL method in Brandt’s voles hole detection in a typical steppe ecosystem. We collected datasets in two different seasons: summer and winter, then investigated six DL-based object detection models, including three two-stage detectors and three single-stage detectors, to explore their accuracy, speed, and generalizability in BV hole detection.

## 2 Study area and data

**Figure 1:**
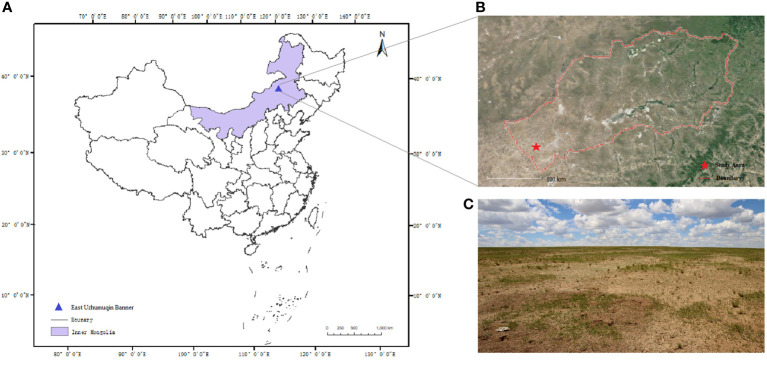
**(A)** Study area in Inner Mongolia, China. **(B)** East Uzhumuqin Banner in Xilingol League. **(C)** The typical steppe where UAS imagery was collected.

Study areas (**Figure 1**) are located in East Uzhumuqin Banner (45°31′0″ N, 116°58′0″E) in Xilingol League, which is in the northeastern region of Inner Mongolia in China. Xilingol League is the main steppe habitat for Brandt’s vole (others are the Hulunbeir League of China, the Republic of Mongolia, and the Baikal Lake region of Russia) (Zhong et al., 2007). Rodent infestation occurs annually in Xilingol League and is associated with drought and ecological deterioration. In Xilingol Leaure, East Uzhumuqin Banner is the severely damaged region, with a total of 950 km^2^ area affected in 2021, where BV is the primary pest.

**Figure 2:**
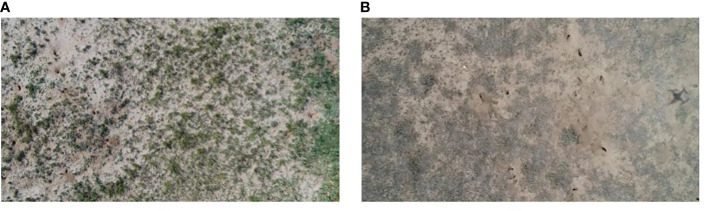
UAS photos captured in summer **(A)** and winter **(B)**.

The experiment was implemented in a typical steppe in East Uzhumuqin Banner. As shown in **Figures 2A, B**, environmental conditions, especially grass conditions, are different between the two seasons. Images were collected in the same pasture in summer (September 9th-12th) and winter (November 1st-5th) in 2020. Both selected seasons have ecological significance. Brandt’s voles reproduce from March to August. The population of the species peaks in September (Shi, 2011). In November, BV holes start clustering for the winter. Most holes become inactive and filled by soil, stones, grass, and snow, thus disappearing. The number of BV holes reaches the lowest in winter and performs as the population baseline for the next year (Wan et al., 2006). Therefore, detection results in September can represent the magnitude of the BV disaster of the current year, and detection in November can help to determine the peak number of rodents in the next year. Images collected in September and November were used to generate two datasets (Dataset1 and Dataset2, respectively). Moreover, we combined these two datasets to generate Dataset3 as a comprehensive dataset. Detailed information about datasets is listed in **Table 1**.

**Table 1:** Datasets information.

	Dataset1	Dataset2	Dataset3
Collection data	November 1st to 5th	September 9th to 12th	The combination of Dataset1 and Dataset2
UAS devices	DJI Inspire 2 + DJI Zenmuse X5S professional gimbal camera+DJI 15mm Micro Four Thirds lens	DJI Inspire 2 + DJI Zenmuse X5S professional gimbal camera+Olympus M.Zuiko 45mm/1.8 lens	
Flight altitude	8 meter	15 meter	
Patch size	500*500	1000*1000	
Patch numbers	2587	2218	4805
BV hole numbers	3412	3279	6691

Flight and data collection was conducted during the whole day from 8 am to 5 pm. The UAS utilized in this study to capture BV hole images was a DJI Inspire 2 equipped with a DJI Zenmuse X5S professional gimbal RGB camera. A DJI 15mm Micro Four Thirds lens and an Olympus M.Zuiko 45mm/1.8 lens were used to capture RGB images in summer and winter, respectively. In preliminary experiments, we explored different flight heights in the detection. We found that a BV hole can be recognized when its bounding box covers at least 30*30 pixels. Therefore, we chose 8m and 15m as the flight heights for the two UAS devices to ensure sufficient spatial resolution. Also, the vertical shooting angle was pre-determined for ortho rectification and mosaicking of images. Images captured by the two lenses have the same size (5280*3956 pixels).

## 3 Methods

**Figure 3:**
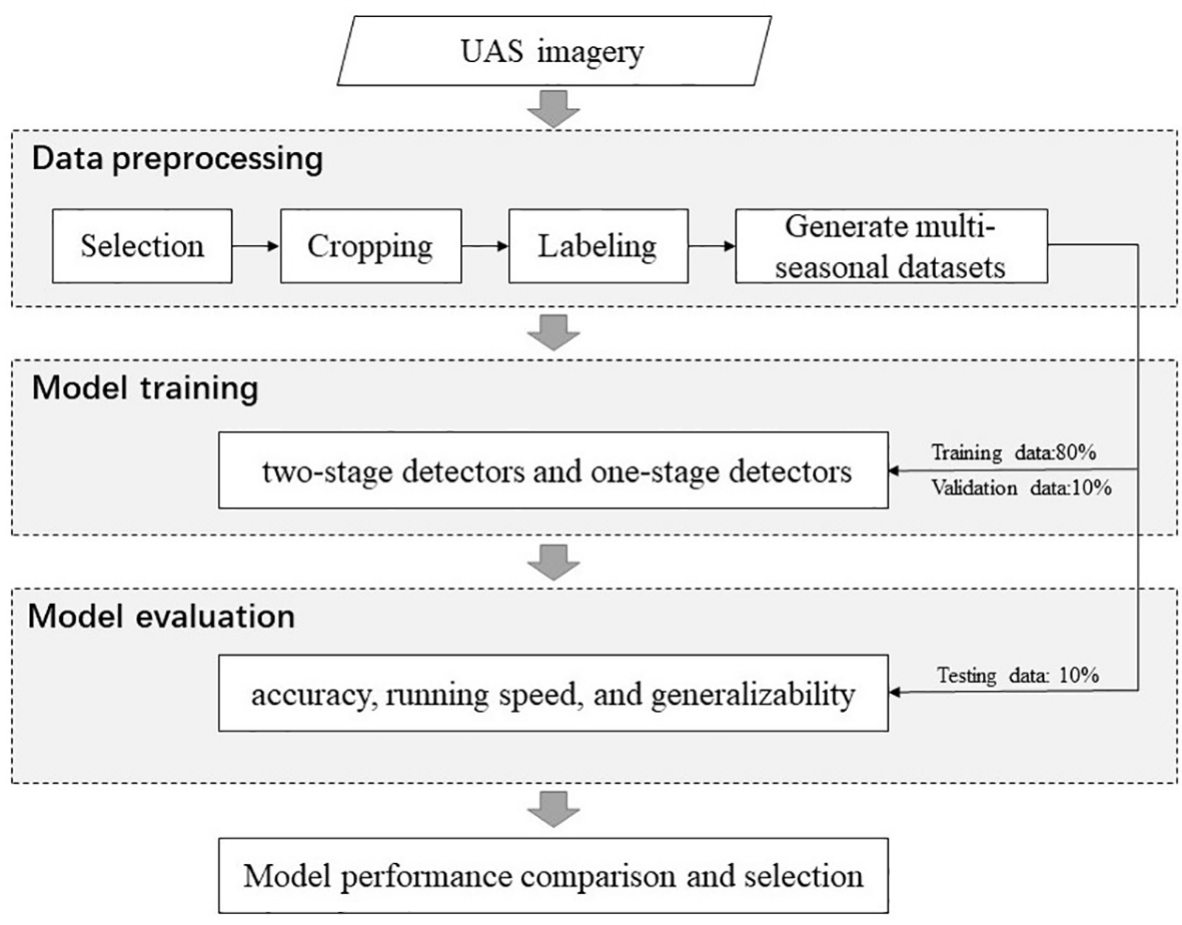
Workflow of this study.

The main method of this study is a supervised object detection approach (**Figure 3**). First, UAS images were collected in two different seasons and preprocessed. Two seasonal datasets and one combination dataset are generated, respectively. Next, to determine the optimal DL model in BV’s hole detection, we trained six representative object detection DL models, including three one-stage and three two-stage models. Finally, the performance of the models was assessed and compared. Detailed methods were presented in Sections 3.1 to 3.4.

### 3.1 Data preprocessing

In this section, raw images captured by UAS were preprocessed through selection, cropping, and labeling. First, images that contained BV holes were selected by experts to filter out invalid data. Then, full scenes of UAS images were cropped into fixed-size patches for further approach regarding computing memory limitation. Dataset1 and Dataset2 were manually cropped into patches with 500*500 pixels and 1000*1000 pixels, respectively. Overlaps between patches were avoided. Third, we labeled BV holes in the patches in LabelImg (Tzutalin, 2016), which is an open-source graphical image annotation tool, by delineating the bounding boxes of BV holes. Every BV hole sample in patches was selected and double-checked by experts. A total of 4805 images with 6691 BV holes were manually annotated for the training, validation, and test datasets. Before training, we implemented data augmentation to extend the datasets, including random scaling, random flipping, random cropping, and hue-saturation-value transformation.

### 3.2 Model training

For each dataset, patches were randomly split into three parts: 80% for training, 10% for validation, and 10% for testing. The training set was used to train the deep learning model; the validation set was used to validate the adopted improving tactics; the testing set was used to evaluate the performance of trained deep learning models.

#### 3.2.1 Deep learning models

We tested six commonly used DL models, which can be grouped into two categories: two-stage detectors and one-stage detectors. Two-stage detectors, e.g., Faster R-CNN, Region-based Fully Convolutional Network (R-FCN), and Cascade R-CNN, conduct region proposal generation and object classification using two different networks. Alternatively, one-stage detectors, e.g., Single Shot MultiBox Detector (SSD), RetinaNet, and YOLOv4, treat object detection as a simple regression problem, thus running the above operations only using one network. Compared with two-stage detectors, one-stage models usually achieve lower detection accuracy but much faster speed. To determine an optimal method that can achieve a balance between accuracy and speed in detecting BV holes from the UAS images, we investigated three representative models from each category. The brief descriptions of models are presented below.

Three two-stage models are Faster R-CNN, R-FCN, and Cascade R-CNN. Faster R-CNN is a classical region-based deep detection model proposed in 2015 (Ren et al., 2015). Faster R-CNN generates feature maps using the deep residual network ResNet-101, which contains 101 convolutional and pooling layers, proposed by He et al. (2016). In the first stage, a Region Proposal Network (RPN) narrows the number of candidate object locations to a small number (e.g., 1~2k) by filtering out most background samples. In the second stage, the proposals from the first stage get features of equal size through Region of Interest (RoI) pooling and are sent to the classifier. After being classified into specific classes, the final object detection results will be provided with more accurate locations *via* bounding-box regression. This model employed a fully convolutional network, which simultaneously predicts object bounds and objectness scores at each position. It truly realized end-to-end training by introducing the basis of Fast R-CNN, which greatly improved the detection speed and accuracy (Girshick, 2015).

R-FCN is a fast approach in the two-stage approach category (Dai et al., 2016; Tsang, 2019). R-FCN also adopted ResNet-101 as the feature extractor. An RPN proposes candidate RoIs, which are then applied on the score maps, using a bank of specialized convolutional layers as the output. The use of position-sensitive score maps addressed the dilemma between invariance/variance on translation. All learnable layers are convolutional and are computed on the entire image. The architecture of R-FCN enables nearly cost-free region-wise computation and speeds up training and inference. It has achieved competitive results with a significantly faster detection speed than the Faster R-CNN.

Cascade R-CNN is a multi-stage object detection algorithm released at the end of 2017 (Cai and Vasconcelos, 2018; Lin et al., 2014). ResNet-101 is also used as the feature extraction backbone in this model. Different from other models, in Cascade R-CNN, increasing thresholds of Intersection over Union (IoU), which is an indicator to judge the degree of overlap between predictions and labels, are trained in multiple cascaded detectors. The cascaded detectors were trained sequentially, where deeper stages are more sensitive against close false positives (Razavi et al., 2021). The inference speed after cascade may be slightly slower but within acceptable limits. Cascade R-CNN is conceptually straightforward, simple to implement, and can be combined, in a plug-and-play manner, with many detector architectures.

Three one-stage models are SSD, RetinaNet, and YOLOv4. SSD uses a set of predefined boxes of different aspect ratios and scales to predict the presence of an object in a certain image (Liu et al., 2016; Soviany and Ionescu, 2018). Particularly, it utilizes different target sizes to extract feature maps and encapsulates all computations in a single network. This design makes SSD easy to train and faster than two-stage models. VGG-16 was employed in this model for feature extraction.

RetinaNet is a one-stage detector that can achieve comparable accuracy to some two-stage models by using focal loss to solve the foreground-background class imbalance problem (Lin et al., 2017). ResNet-101 is used in feature extraction. A Feature Pyramid Network (FPN) is proposed to construct a multi-scale feature pyramid from one single-resolution input image. RetinaNet is multi-scale, semantically strong at all scales, and fast to compute.

YOLOv4,the fourth version of YOLO, is a widely used, state-of-the-art, real-time object detection system (Redmon et al., 2016; Zou, 2019). YOLOv4 was proposed in 2020, which used novel CSPDarknet53 as a backbone and added universal algorithms, e.g., DropBlock Regularization. The Spatial Pyramid Pooling block was added over the CSPDarknet53 to increase the receptive field of the backbone features and separates the most significant context features. Instead of the FPN used in YOLOv3, PANet was used as the method of parameter aggregation from different backbone levels for different detector levels. Benefiting from the novel backbone and new features, YOLOv4 has enhanced learning capability and improved detection accuracy while assuring its positioning speed compared with YOLOv3. It also became easier to train on a single GPU (Bochkovskiy et al., 2020).

#### 3.2.2 Model hyper-parameter settings

Model hyper-parameters, i.e., learning rate, batch size, iterations, and epochs, were adjusted during training. All models are trained with the Stochastic Gradient Descent (SGD) algorithm, and the optimal values of these hyper-parameters are listed in **Table 2**. At the end of the training, the validation loss reached a convergence state for all six models. The experiment was implemented on NVIDIA Tesla P100 GPU with an Inter(R) Xeon(R) Gold 6132 CPU with 16 G RAM. All the methods were implemented in PyTorch.

**Table 2:** Model hyper-parameters settings.

Models	Feature extraction network	Batch size	Learning rate	Iterations	Epochs
**Faster R-CNN**	ResNet-101	64	0.001	50000	1282
**R-FCN**		64	0.001	200000	5128
**Cascade R-CNN**		64	0.001	200000	5128
**RetinaNet**		16	0.001	46875	300
**SSD**	VGG16	32	0.001	120000	3096
**YOLOv4**	CSPDarknet53	16	0.001	46875	300

### 3.3 Model evaluation

Nine indicators, including True Positive (TP), False Positive (FP), True Negative (TN), False Negative (FN) (**Table 3**), Recall, Precision, Average Precision (AP), F1-score, Average AP, Average F1-score, and Frames Per Second (FPS) were utilized to evaluate the performance of models.

**Table 3:** The confusion matrix for the possible outputs.

Actual class	Predicted class	
	Positive	Negative
**Positive**	TP	FN
**Negative**	FP	TN

TP is an outcome where the model correctly predicts the positive class. Alternatively, TN is an outcome where the model correctly predicts the negative class. FP is an outcome where the model incorrectly predicts the positive class, and FN is an outcome where the model incorrectly predicts the negative class. True or false was determined by the threshold of intersection over union (IoU). IoU measures the overlap ratio between the detected object (marked by a bounding box) and the ground truth (an annotated bounding box). The threshold was set to 0.5, which means that a detection result is determined as true when IoU>=0.5.

We use *Recall* and *Precision* to evaluate the predictability of the BV hole detection model. *Recall* presents the ability to find all relevant instances in a dataset (Equation 1), and *Precision* presents the percentage of the instances which are correctly detected (Equation 2).


(1) $$Recall=\frac{TP}{TP+FN}\times 100\%$$

(2) $$Precision=\frac{TP}{TP+FP}\times 100\%\;$$

AP and F1-score were employed to comprehensively evaluate the results since *Recall* and *Precision* reflect only one aspect of the model’s performance. *AP* (Equation 3) is the area under the curve of *Precision* and *Recall* rate, which is an intuitive evaluation standard for the model accuracy and can be used to analyze the detection effect of a single category. F1-score (Equation 4) is the harmonic mean of precision and recall.


(3) $$AP=\sum_{i=1}^{n}Precision_{i}\left(Recall_{i}-Recall_{i-1}\right),\;with\;Recall_{i=0}=0$$

(4) $$F1-score=\frac{2\times Recall\times Precision}{Recall+Precision}$$

In addition, the Average AP and Average F1-score are the arithmetic mean of the APs and F1-scores among prediction results in all three datasets.

In addition, FPS (Equation 5) is used to assess the model efficiency. In FPS calculation, 150 patches act as the input of the trained model to obtain the T (total running time). FPS can be calculated by Equation 5 (it usually takes more time for the first picture to load the model, so the time of the first picture is not counted). Higher FPS indicates higher speed and better efficiency.


(5) $$FPS=\frac{149}{T}$$

## 4 Results

### 4.1 Results from different models

**Figure 4:**
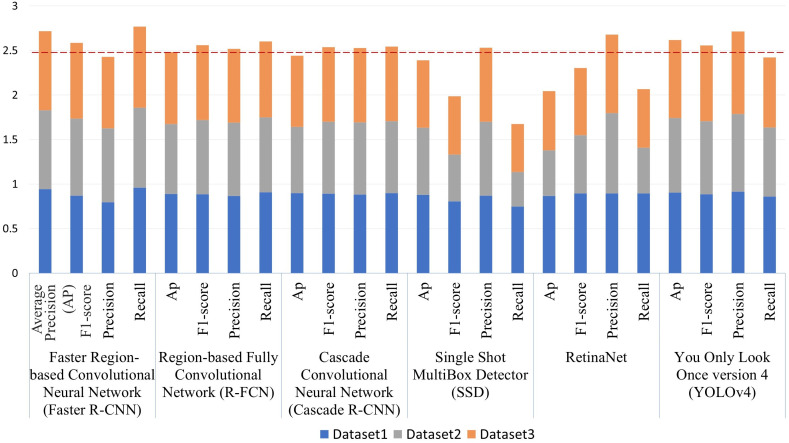
Detection accuracy of six different Deep-learning (DL) models using three datasets. The red dashed line is the mean (2.448) of the sum of the Average Precision (AP) of six DL models: Faster Region-based Convolutional Neural Network (Faster R-CNN), Region-based Fully Convolutional Network (R-FCN), Cascade Convolutional Neural Network (Cascade R-CNN), Single Shot MultiBox Detector (SSD), You Only Look Once version 4 (YOLOv4).

Six DL object detection models were compared through the experimental results (**Table 4**). In terms of accuracy, the accuracies of the two-stage models were higher than those of one-stage models, except YOLOv4. Faster R-CNN achieved the highest accuracy with 0.905 in Average AP, seconded by YOLOv4 (0.872). RetinaNet had the lowest Average AP (0.681). Regarding the Average F1-score, Faster R-CNN also had the highest value, 0.86, followed by R-FCN (0.853) and YOLO v4 (0.852). SSD had the lowest Average F1-score, which is 0.662. As shown in **Figure 4**, Faster R-CNN and YOLOv4 achieved the best accuracies combining all datasets.

**Table 4:** Accurcies and speed of different models.

Categories	Models	Average AP	Average F1-score	FPS
Two-stage	Faster R-CNN	0.905	0.861	4.62
	R-FCN	0.827	0.853	6.86
	Cascade R-CNN	0.814	0.845	2.81
One-stage	SSD	0.800	0.662	29.14
	RetinaNet	0.681	0.767	7.17
	YOLOv4	0.872	0.852	10.62

In terms of speed, the running speed of the two-stage models was significantly slower than that of the one-stage. SSD uses a shallow VGG-16 network as its backbone and had the fastest running speed, 29.14 frames/second. YOLOv4 using CSPDarknet53 follows, which had 10.62 frames/second. The FPS of other models used the ResNet-101 network as backbones were all below eight frames/second.

### 4.2 Results from seasonal datasets

**Figure 5:**
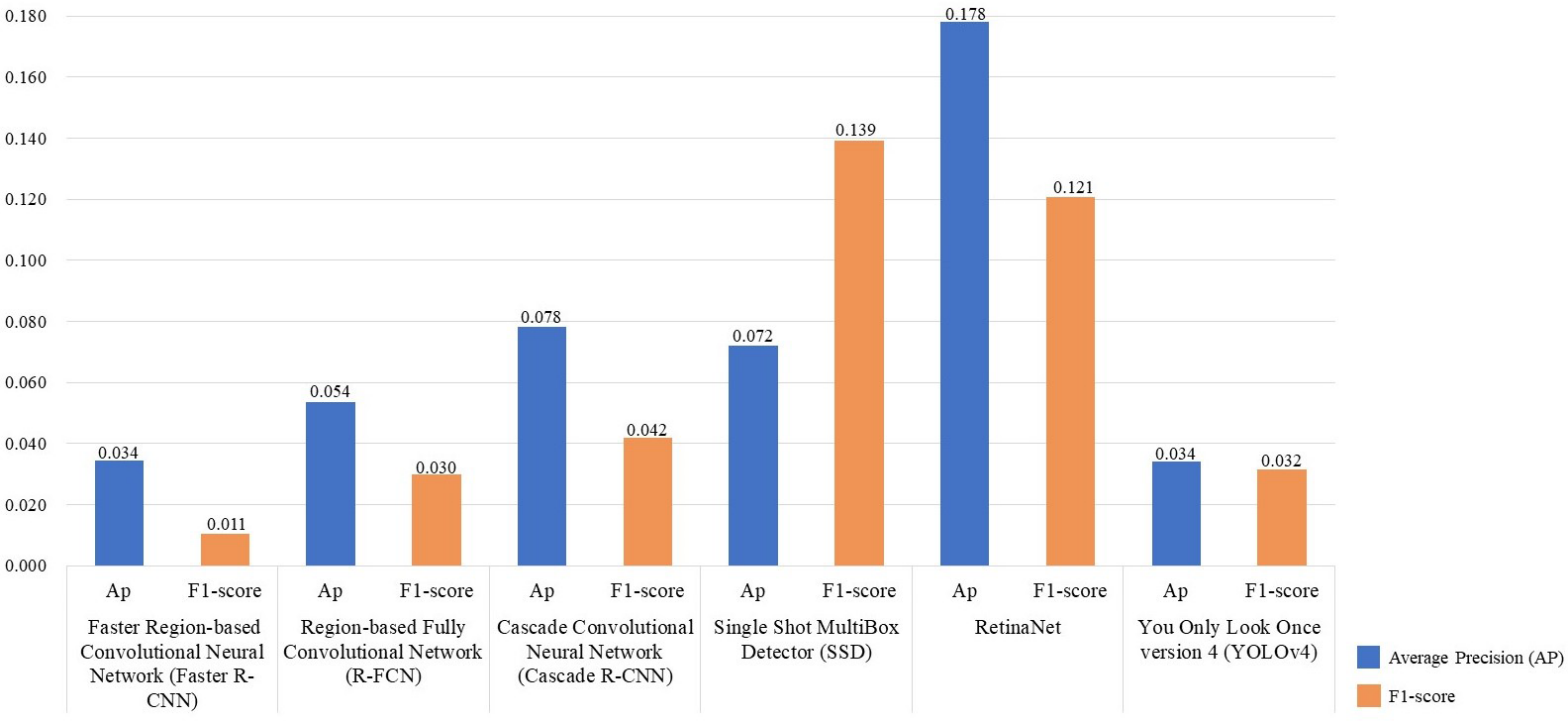
The standard deviation of Average Precision (AP) and F1-score in three datasets for six Deep-Learning (DL) models: Faster Region-based Convolutional Neural Network (Faster R-CNN), Region-based Fully Convolutional Network (R-FCN), Cascade Convolutional Neural Network (Cascade R-CNN), Single Shot MultiBox Detector (SSD), You Only Look Once version 4 (YOLOv4). The standard deviation measures the dispersion of accuracy among three datasets.

We also compared the models’ performance among different datasets collected in two seasons (**Table 5**). Generally, all results from different models and datasets had acceptable accuracy, most of which had above 65% AP and F1-score. We performed a t-test for each pair of datasets. Detection results in early winter (Dataset1) were more accurate than those in summer (Dataset2), considering their AP and F1-score were significantly different at the 10% confidence level. In addition, based on the statistical test results, detection accuracy using Dataset3 was significantly lower than using Dataset1 and higher than using Dataset2. Among all models, Faster R-CNN and YOLOv4 were the two best models in terms of generalizability, regarding their low standard deviation of AP and F1-score among the three datasets (**Figure 5**).

**Table 5:** The performances of different models using three datasets.

Models	Datasets	True number	Predicted number	Precision	Recall	Ap	F1-score
Faster R-CNN	Dataset1	354	427	0.796	0.961	0.945	0.871
	Dataset2	321	346	0.832	0.897	0.884	0.863
	Dataset3	646	735	0.799	0.909	0.887	0.850
R-FCN	Dataset1	354	370	0.868	0.907	0.888	0.887
	Dataset2	321	329	0.821	0.841	0.786	0.831
	Dataset3	646	665	0.829	0.853	0.808	0.841
Cascade R-CNN	Dataset1	354	360	0.883	0.899	0.898	0.891
	Dataset2	321	317	0.811	0.807	0.743	0.809
	Dataset3	646	651	0.833	0.839	0.801	0.836
SSD	Dataset1	354	305	0.869	0.749	0.880	0.805
	Dataset2	321	149	0.832	0.386	0.753	0.527
	Dataset3	646	419	0.831	0.539	0.757	0.654
RetinaNet	Dataset1	354	354	0.895	0.895	0.867	0.895
	Dataset2	321	183	0.901	0.514	0.512	0.655
	Dataset3	646	482	0.881	0.656	0.663	0.752
YOLOv4	Dataset1	354	333	0.913	0.859	0.905	0.885
	Dataset2	321	285	0.874	0.776	0.837	0.822
	Dataset3	646	548	0.925	0.785	0.874	0.849

## 5 Discussion

In this paper, we developed the first Brandt’s voles hole detection method that can be utilized in a typical steppe ecosystem using UAS and DL. We established the first BV hole UAS image dataset, including samples in summer and winter. Detection results from six popular DL models were explored and compared. To our knowledge, this is the first UAS-based hole detection study for the specific species, *Lasiopodomys brandtii*. Advantages, findings, and limitations have been discussed below.

### 5.1 Advantages of UAS and DL models

Generally speaking, DL models based on UAS imagery had satisfactory results in BV hole detection. For example, using the model of Faster R-CNN and YOLOv4 to detect BV holes in UAS images, we can achieve a high Average AP, i.e., 0.905 and 0.872, which is a compelling output. More importantly, the proposed approach significantly improved the efficiency of the investigation. UAS-DL-based methods took less time and labor than traditional field survey methods. Specifically, taking the 0.25hm^2^ plot (the commonly used size for a manual survey plot) as an example, traditional manual methods require five or six people to spend about 1 hour. Repetitive counting in traditional methods may lead to a huge margin of error. In addition, human trampling during the investigation may cause destructive damage to grasslands. Therefore, it is not suitable for large-scale and periodic repeated monitoring. In monitoring by UAS, the aerial photography acquisition requires only one person and takes about 15 minutes for the same area (0.25hm^2^), which greatly improves the survey efficiency without damage. The running speed of SSD and YOLOv4 can achieve the FPS of 29.14 frames/second and 10.62 frames/second. For example, the proposed method using YOLOv4 needs only about 10 minutes for a 0.25hm^2^ plot to obtain the detection result. To this end, the proposed framework of UAS and DL models is an effective and efficient method for identifying BV holes.

### 5.2 Model comparison and selection

We compared the models from the following three perspectives: accuracy, running speed, and generalizability.

**Figure 6:**
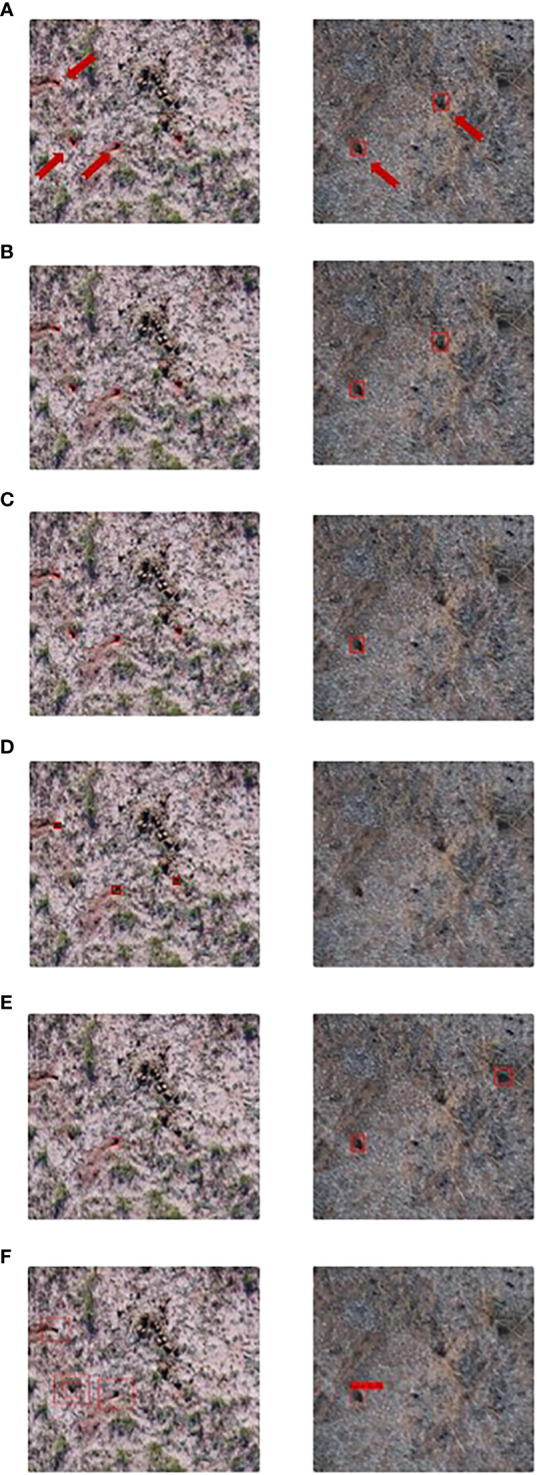
Test samples of BV hole detection using Deep-Learning (DL) algorithms in UAS images (left: summer samples, right: winter samples). **(A)** Faster R-CNN, **(B)** Cascade R-CNN, **(C)** R-FCN, **(D) **SSD, **(E)** RetinaNet, and **(F)** YOLOv4. Predicted BV holes are in red circles. Actual holes are pointed out using red arrows **(A)**.

From the accuracy perspective, as shown in **Table 4**, Faster R-CNN and YOLOv4 were the two most accurate models with the highest average AP (0.904 and 0.872). It also should be noted that Faster R-CNN had the highest recall (0.961) while its precision was relatively low (0.796), which indicates that Faster R-CNN can detect more BV holes but may contain more false detections (**Figure 6**). YOLOv4 had the highest precision (0.913) and an acceptable recall (0.859).

From the running speed perspective, SSD and YOLOv4, which used VGG16 and CSPDarknet53 as their feature extraction networks, were the two fastest models with the highest FPS (29.14 and 10.62). All other models were used the ResNet-101 network as backbones, which has more parameters, thus required longer running time. However, SSD was excluded in practical applications since it had the lowest Average F1-score (0.662).

From the generalizability perspective, we focused on accuracy stableness, which refers to the variance of accuracy among different datasets. As mentioned in section 4.2, all models were performed better in winter than summer (**Table 5**). The main reason for this was that grass withers in winter, which leads to a much clearer view field. Less occlusion from grass in winter will lead to fewer missing BV holes in the detection. Additionally, fewer shadows and shades in winter will result in fewer FP.

Specifically, we discussed the advantages and disadvantages of each model one by one based their performance. Faster R-CNN had the highest accuracy considering AP and F1-score but relatively lower running speed. The RPN used to select candidate objects in the first stage of the model can utilize multi-scale feature information that improves performance in detecting small objects, e.g., BV holes. In contrast, much detailed information in RPN results in a long running time. In addition, it could detect the most BV holes among all models but may contain more false detections because fewer negative classes were sent to training after random sampling in RPN. R-FCN had the fastest running speed among two-stage models but a relatively lower Average AP, which indicates that features of BV holes are less sensitive to the problem that R-FCN majorly solved, i.e., the contradiction between translation variance and invariance. Cascade R-CNN had the lowest running speed thanks to its cascade architecture, with only 2.81 frames/second. However, its accuracy did not get satisfactory improvement after the cascade.

The efficiency of the one-stage models was significantly better than two-stage models. Benefiting from the VGG-16 network, SSD had the fastest running speed, which was more than ten times faster than Cascade R-CNN. However, the F1-score of SSD was the lowest, directly caused by the lowest recall due to the insufficient convolutional layers to extract features. Although RetinaNet had a higher F1-score than SSD benefiting from the focal loss function, its Average AP was the lowest among all models. The reason for this could be that RetinaNet pays more attention to difficult samples, thus leading to worse performance in easy and majority samples. YOLOv4 was ranked second both in accuracy and speed. Its one-stage architecture and the novel CSPDarknet53 feature extraction network reduced calculation when maintaining accuracy. In YOLOv4, the receptive field increases, and the size of the feature map decreases as the network deepens. Features and locations become abstract and fuzzy as well. While Faster R-CNN used FPN to construct a multi-scale feature pyramid for small objects, YOLOv4 had lower accuracy than Faster R-CNN in BV hole detection. On the other hand, YOLOv4 had a more balanced performance on missing BV hole detection and false detection than Faster R-CNN due to calculating the confidence loss for all positive and negative samples.

We further explored the generalizability of Faster R-CNN and YOLOv4 in a supplementary experiment. Specifically, we employed Dataset3, which is the most comprehensive dataset, as the training set, and tested the models on single-season datasets separately, i.e., Dataset1 and Dataset2. Experimental results showed that when using a more comprehensive training dataset in Faster R-CNN, the detection accuracy was significantly improved in both summer and winter (**Table 6**). Alternatively, when using YOLOv4, the detection accuracy was improved only in the winter dataset but decreased in the summer dataset. Furthermore, the accuracy improvement by Faster R-CNN was higher than by YOLOv4. To conclude, Faster R-CNN had a more accurate and consistent performance in two seasons.

**Table 6:** The performance in the generalizability test using different models.

Models	Training dataset	Testdataset	True number		Predicted number	Preci-sion	Recall	Ap	F1-score
**Faster R-CNN**	Dataset3	Dataset1		354	411	0.867	0.983	0.977	0.921
	Dataset3	Dataset2		321	371	0.841	0.970	0.951	0.901
**YOLO-v4**	Dataset3	Dataset1		354	328	0.940	0.870	0.937	0.903
	Dataset3	Dataset2		321	420	0.662	0.867	0.760	0.750

In summary, when considering accuracy, Faster R-CNN and YOLOv4 were the two best models; when considering running speed, SSD and YOLOv4 were preferred; when considering generalizability, both Faster R-CNN and YOLOv4 were achieved acceptable accuracy in two seasons. Therefore, if taking into account accuracy, speed, and generalizability, the YOLOv4 was the best choice.

### 5.3 Uncertainty and limitations

**Figure 7:**
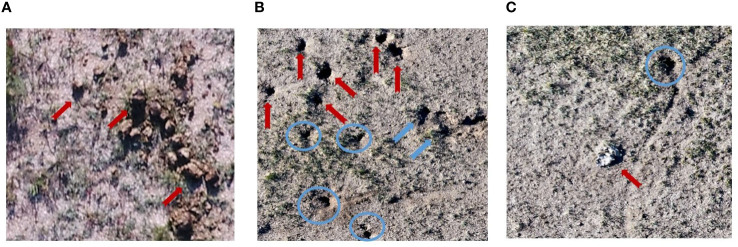
Noises in detection in a typical steppe ecosystem are pointed out using red arrows. **(A)** Animal droppings, **(B)** hoofprints (red arrows) and shadows of grass (blue arrows), **(C)** shadows of rock. Actual BV holes are in blue circles.

**Figure 8:**
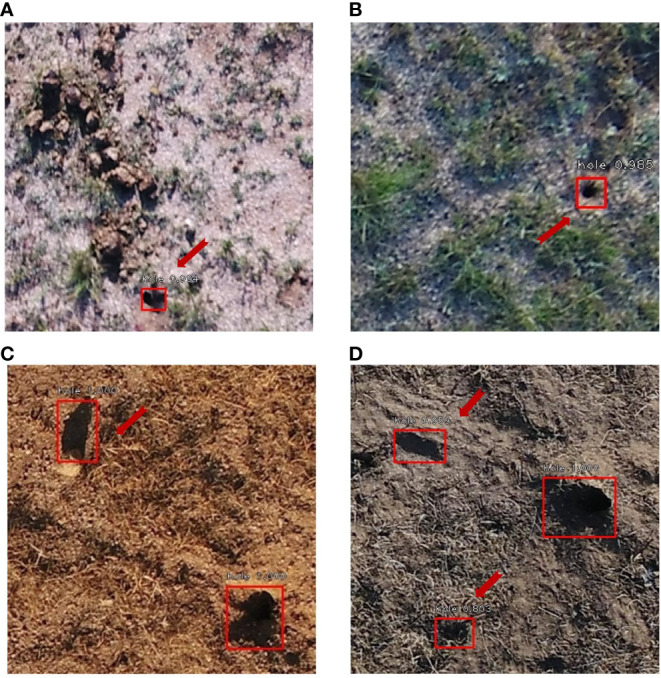
Examples of False Positive (FP) (red arrows): **(A)** cow dung, **(B)** shadows of grass, **(C)** shadows of rocks (the upper left), and **(D)** shadows of rocks (the upper left) and grass (the lower left). Predicted BV holes are in red boxes.

**Figure 9:**
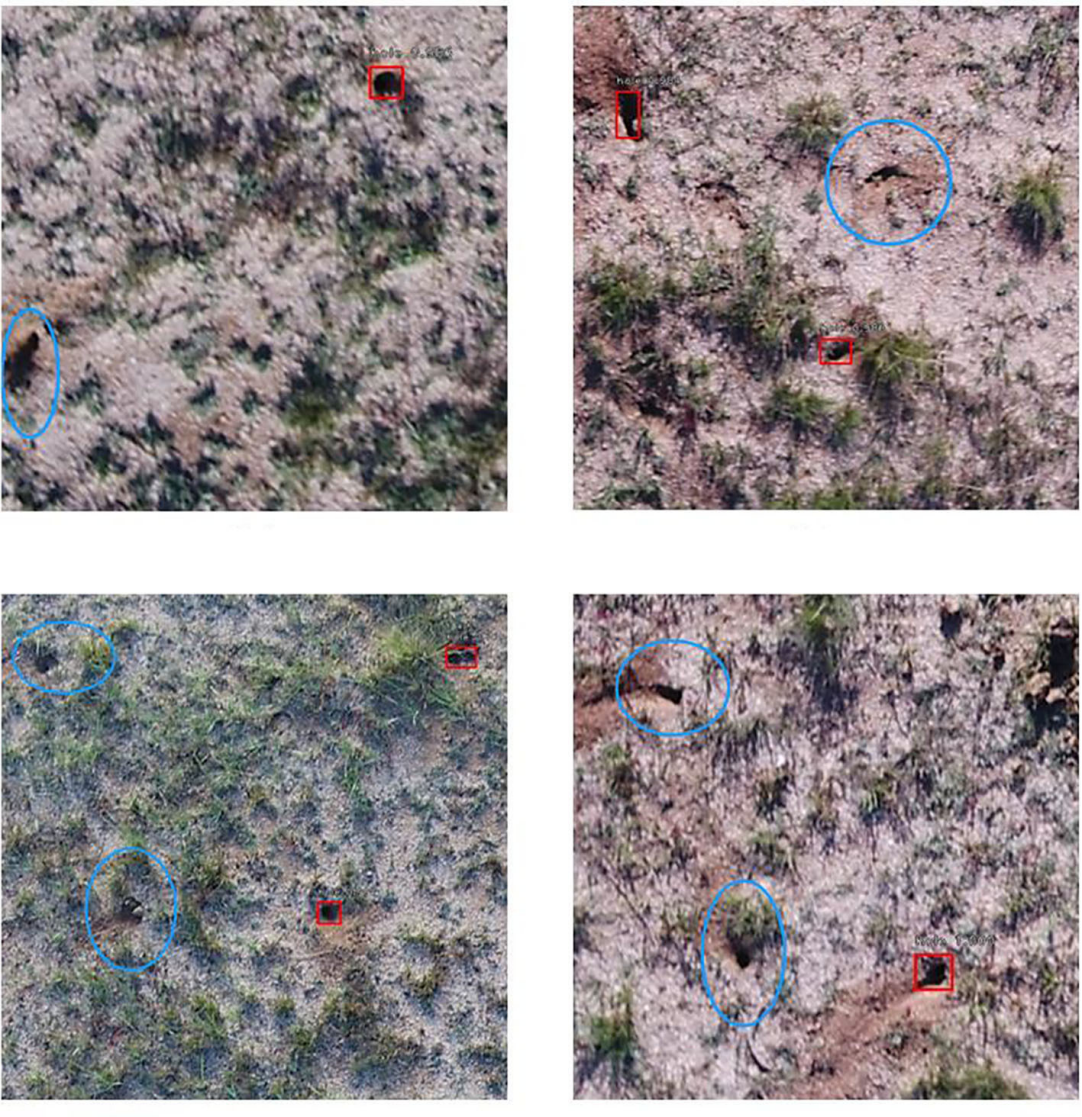
Examples of False Negative (FN) (in blue circles). Predicted BV holes are in red boxes.

There were several sources of uncertainty in the proposed UAS-DL-based BV hole detection method. First, noises like animal droppings, hoofprints, and shadows of grass and rocks were frequently appeared within the UAS images (**Figure 7**). Most of the falsely detected BV holes came from the misclassification of these noises (**Figure 8**). Second, various shapes and sizes of BV holes were brought uncertainty and error in the detection. In the complex wild environment, the shape and size of rodent holes depend on various factors, e.g.,the size of the rodents that live in, the rodents’ activity level, and the erosion degree. The lack of outlier-shaped samples lead to many FNs (**Figure 9**).

The limitations of this study are presented as follows. First, due to the top-view perspective, BV holes occluded by other objects cannot be detected from UAS. Second, small target detection still needs to be improved, especially in complex environments, e.g., typical steppe ecosystems. More advanced algorithms may improve the accuracy and consistency of BV hole detection. Besides the abovementioned future directions, real-time and large-scale BV detection studies are essential for better rodent monitoring. Moreover, spatial distribution and surrounding environment analysis are worthy of further exploration, which can provide more valuable advice on rodent disaster management and grassland protection.

## 6 Conclusions

A UAS-DL-based BV hole detection framework that can be used in different seasons was developed in this study. After comparing different DL models’ accuracy, speed, and generalizability in bi-seasonal datasets, we suggested an optimal model, YOLOv4, for BV hole detection in typical steppe ecosystems. In addition, we established a bi-seasonal BV hole UAS image dataset. To our knowledge, this is the first study that employs UAS images in BV hole detection. Furthermore, the seasonal effect was first considered and solved in rodent hole detection studies. The suggested model and dataset have a great potential for large-scale multi-temporal rodent hole detection and better management by the grassland ecological protection departments.

## Data availability statement

The raw data supporting the conclusions of this article will be made available by the authors, without undue reservation.

## Author contributions

MD, DW and SL initiated the research idea and designed and conducted the experiments. MD finish the writing of this manuscript with the assistance of DW. DW and SL provided the financial and equipment support to make this study possible. CL and YZ provided important insights and suggestions on this study from the perspective of algorithms and plant protection experts. All authors contributed to the article and approved the submitted version.
